# Changes in Chinese Policies to Promote the Rational Use of Antibiotics

**DOI:** 10.1371/journal.pmed.1001556

**Published:** 2013-11-19

**Authors:** Yonghong Xiao, Jing Zhang, Beiwen Zheng, Lina Zhao, Sujuan Li, Lanjuan Li

**Affiliations:** Collaborative Innovation Center for Diagnosis and Treatment of Infectious Diseases, State Key Laboratory for Diagnosis and Treatment of Infectious Diseases, The First Affiliated Hospital, College of Medicine, Zhejiang University, Hangzhou, China

## Abstract

Xonghong Xiao and colleagues analyze the challenge of antimicrobial resistance in China. A government strategy to promote rational use of antimicrobials in health care reduced antibiotic sales and percentage of prescriptions for antimicrobials for both hospitalized patients and outpatients, and offers insights to shape future initiatives.

*Please see later in the article for the Editors' Summary*

**Summary Points**

- Microbial resistance to antimicrobial agents (antimicrobial resistance, AMR) is a serious public health challenge, and containment of AMR is an urgent priority, both in China and worldwide.
- The main cause of AMR is the irrational use of antimicrobial agents, in healthcare and veterinary settings and by the general public. Actions taken by the Chinese health administrative authorities in the past 10 years have been largely unsuccessful, likely because of the lack of mandatory regulations.
- In 2011, coupled with new healthcare reforms, the Chinese Ministry of Health changed strategy and launched a special campaign to promote the rational use of antimicrobials in healthcare settings. This mainly consisted of establishing mandatory management strategies, such as target setting, taskforce organization, and the development of audit and inspection systems.
- The special campaign had notable achievements, with decreased antibiotic sales and a reduced percentage of prescriptions for antimicrobials for both hospitalized patients and outpatients.
- A number of issues still need to be addressed to ensure further improvements in AMR containment. These include the unregulated use of antibiotics in animal husbandry, over-the-counter purchases of antibiotics, and elimination of economic incentives for drug sales.

Antimicrobial resistance (AMR) is a serious public health challenge and containment of AMR is a global priority. On World Health Day in April 2011, the World Health Organization appealed to all member countries to “combat drug resistance: no action today, no cure tomorrow” [1]. The implementation of new Chinese policies over the past 2 years for the rational use of antimicrobials and AMR containment is a promising response to this appeal.

## The Causes of Increasing Antimicrobial Resistance in China

**Figure 1:**
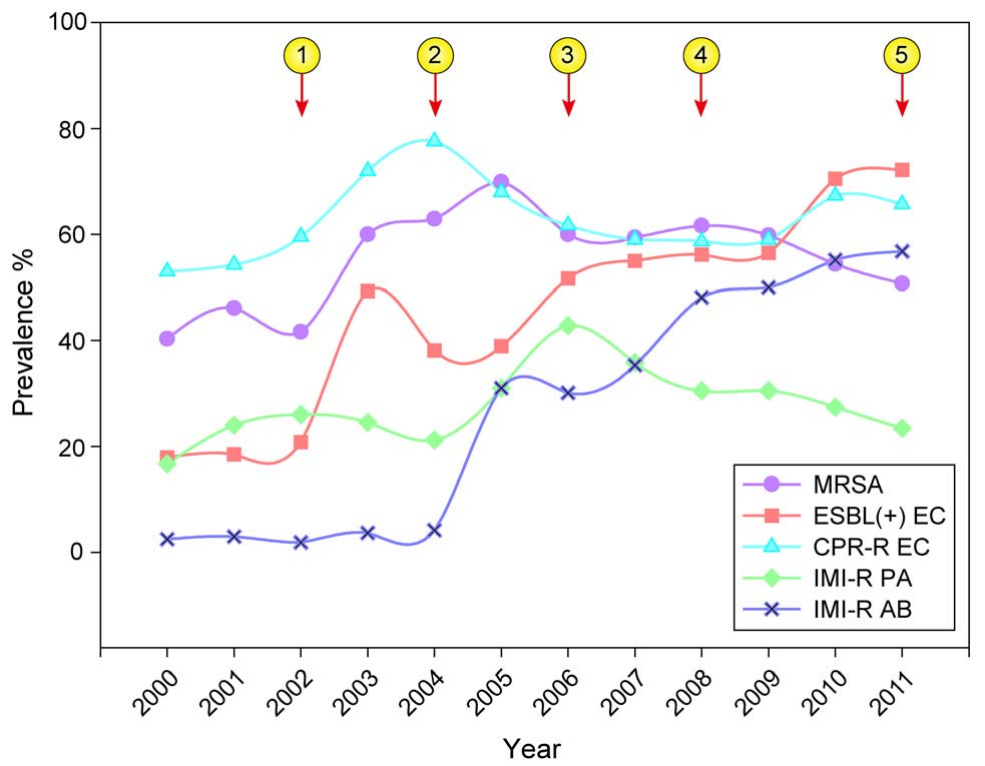
Overall trends in prevalence of major antimicrobial-resistant bacteria in Chinese tertiary hospitals in 2000–2011. The majority of the data were adapted from Mohnarin results, which mostly represent situations involving nosocomial infections in tertiary hospitals. The numbers in circles describe the chronology of major administrative interventions taken by the Chinese Ministry of Health. 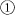 indicates the issue of “temporary rules for pharmaceutical affairs in healthcare institutions” (2002); 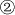 indicates the issue of “guidance for the clinical use of antimicrobials” (2004); 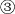 indicates the issue of “regulations for management of nosocomial infections' (2006); 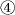 indicates the issue of recommendations for enhancing the prevention and control of multidrug resistant bacterial infections (2008); and 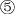 indicates the special campaign initiated in 2011. MRSA, methicillin-resistant *Staphylococcus aureus*; ESBL (+) EC, extended-spectrum β-lactamase-producing *Escherichia coli*; CPR-REC, ciprofloxacin-resistant *E. coli*; IMI-R PA, imipenem-resistant *Pseudomonas aeruginosa*; IMI-R AB, imipenem-resistant *Acinetobacter baumannii*.

The prevalence of AMR is relatively high in China; the morbidity and mortality from infections caused by multidrug-resistant or pan-drug-resistant pathogens are higher in China than in other countries [2]. Data reported by the Chinese Ministry of Health (MOH) National Antimicrobial Resistance Investigation Net (Mohnarin) indicates that AMR is rising steadily. The prevalence of methicillin-resistant *Staphylococcus aureus* (MRSA), extended-spectrum β-lactamase-producing *Escherichia coli*, imipenem-resistant *Pseudomonas aeruginosa*, and imipenem-resistant *Acinetobacter baumannii*—the so-called “superbugs” in nosocomial infections—was 50.5%, 71.2%, 23.4%, and 56.8% in 2010, respectively (Figure 1). The overall prevalence of erythromycin-resistant *Streptococcus pneumonia* and ciprofloxacin-resistant *E. coli* was 94.7% and 65.7% in community settings, respectively [3],[4].

Irrational use of antimicrobial agents is the main cause of increased AMR. In China, antibiotics are considered to be a panacea by the general public and some healthcare practitioners. Over-the-counter purchase and over-reliance on antibiotics for disease therapy, infection prevention, and animal growth promotion are common phenomena in healthcare settings and veterinary practice. The percentage of prescriptions containing antibiotics in rural clinics of western China was around 50%, which is higher than in developed countries such as the US and Sweden [5]. Another important cause of the irrational use of antibiotics is the financial compensation provided to healthcare institutions for drug sales. The Chinese healthcare system consists of a government-led system with characteristics of free market financing and lower service pricing. The government's contribution to hospital budgets is less than 20% of hospital expenditure. Healthcare institutions can receive financial compensation by selling healthcare services and drugs. The government's acquiescence to the situation has stimulated, and to some extent encouraged, excessive examinations, unnecessary treatment, and overuse of medicines by routine healthcare services. Drug sales constitute about half of institutional income and most of the profit, with more than 25% being sales of antimicrobial agents [6]. At the same time, the overuse of antimicrobial agents in animal husbandry and farming has contributed to the occurrence and spread of antibiotic-resistant microbes in the environment [7],[8].

### The Limited Effect of Professional Strategies for the Rational Use of Antibiotics

To control AMR, the Chinese health administrative authorities have taken a series of actions over the past 10 years, including the development of technical specifications and policies. These included the issue of guidance about antibiotic use and infection control. Mohnarin and the Chinese MOH Center for Antibacterial Surveillance (Mohcas) were established in 2006. Hospitals were required to set up a drug therapeutics committee to facilitate the rational use of drugs, and medical practitioners were required to prescribe antibiotics in a rational manner. Furthermore, nosocomial infection control measures were emphasized, such as hand hygiene and contact precautions for AMR infections (although compliance is often unsatisfactory because of heavy staff workloads and large patient populations). In addition, nationwide continuing medical education programs focusing on rational drug use and AMR control, were conducted repeatedly. All of the measures were established as part of a technical support system to promote the rational use of antibiotics, as recommended by the World Health Organization [9],[10].

However, due to strained resources, insufficient enforcement, absence of supervision and inspection, and inefficient implementation plans, these policies and strategies were not successful. Indeed, during the past decade, antimicrobials have remained the most prescribed institutional medicine, and AMR has continued to increase, in some cases dramatically (for example, imipenem-resistant *Acinetobacter baumannii*) (Figure 1) [3],[11],[12].

## Changes in Policy to Promote the Rational Use of Antibiotics

In 2009, a new round of healthcare reforms was initiated in China with the ultimate target of eradicating the “difficulty and high price of seeing a doctor,” and to achieve basic medical security for everyone by 2020. The government increased healthcare spending and promoted healthcare insurance coverage for urban and rural residents. The aim was for the medical establishment to gradually regain its public service role. All these reforms contributed to policies promoting sustainable progress towards the rational use of antimicrobials [13].

In 2011, coupled with healthcare reforms, the Chinese MOH adjusted the reliance on the professional strategies described above and launched a special campaign to reorganize the rational use of antimicrobials in healthcare settings. The campaign protocol mainly consists of establishing mandatory administrative strategies for the rational use of antimicrobials, setting targets for antimicrobial management, organizing task forces, developing audit and inspection systems, and investigating and reassigning responsibility to hospital management staff who violate rational use policies. According to the protocol, all hospitals should have an antibiotic administrative group chaired by the president, formulary restrictions are to be enforced, prescribers have accredited prescription rights for different antibiotic classes determined by their positional titles, and antibiotic procurement should be restricted to 50 or 35 agents in secondary and tertiary hospitals, respectively. Meanwhile, targets for antibiotic prescription are set at less than 60% and 20% of all prescriptions for hospitalized patients and outpatients, respectively; prophylactic use of antibiotics in clean operations should be lowered to 30% of patients and reduced to less than 24 hours' duration; and antibiotic utilization in hospitalized patients should be less than 40 daily defined doses per 100 patient days. All the indicators are linked to hospital quality evaluation procedures and the allocation of future medical resources. Furthermore, adherence to these protocols are to be considered when appointing or dismissing hospital presidents.

To enact these protocols, the Chinese MOH and local health administration signed an administrative target responsibility agreement with major hospital authorities. The Chinese MOH also held several national medical education programs each year and conducted twice yearly inspections (every September and December). According to the protocols, hospitals that fail to meet targets would be downgraded to a lower classification level, and the leaders of the institutes involved would be dismissed. Medical staff who seriously violate the regulations could lose their accreditation to prescribe antibacterial agents, have their professional qualification revoked, or even be prosecuted if their actions have serious consequences [14].

**Figure 2:**
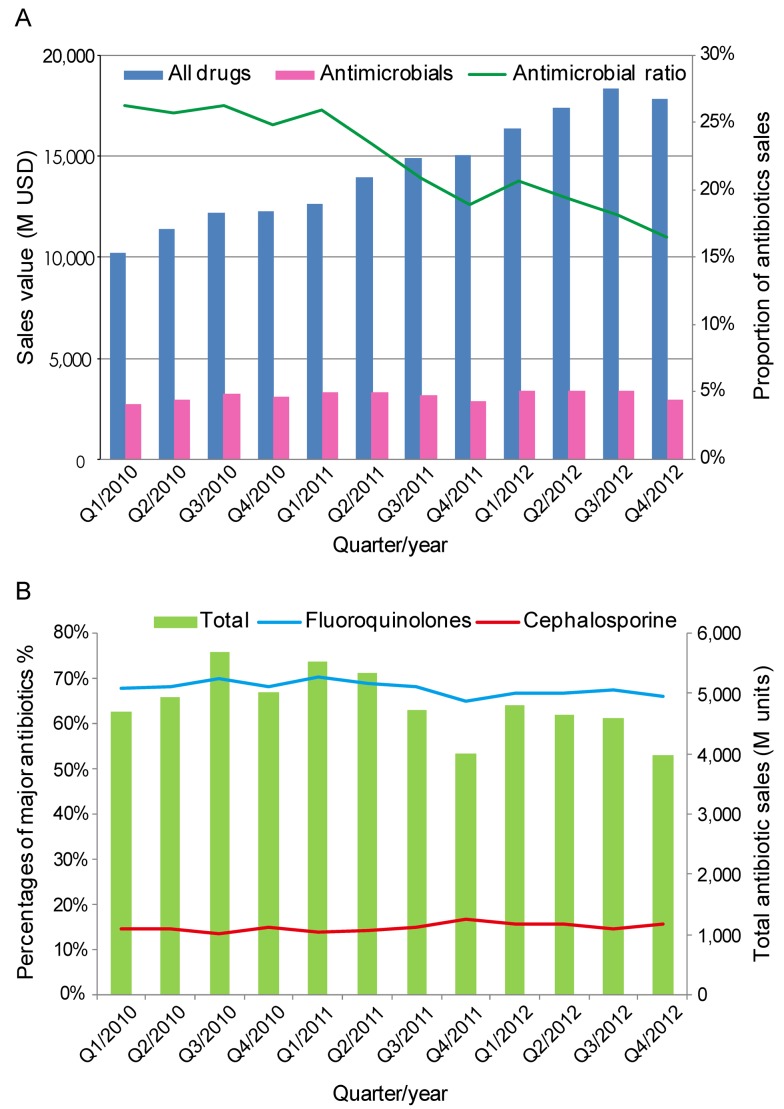
Quarterly sales volume and the proportion of antimicrobial agents sold in 2010–2012 in China. (A) The sales value of all drugs (blue bars) or antimicrobial agents (pink bars) in millions (M) of US dollars (USD) and the proportion of antimicrobial agents sold (green line). (B) The sales volume of all antimicrobial agents in minimal packaging units (green bars) and the percentage of fluoroquinolones (blue line) or cephalosporin sales (red line). Data were adapted from IMS Research.

During the past 2 years in China, these policies were enacted with extensive promotion of education and increased levels of supervision. Excellent hospitals were recognized, and failures were criticized in public. Several hospital presidents were dismissed and some clinicians were punished with economic sanctions. A few clinicians also had their prescription rights suspended by health authorities as a result of severe regulatory violations. A gradual return towards the rational use of antimicrobials has begun. According to IMS Research data, the percentage of drug sales (by value) for antimicrobials decreased to 17% in the fourth quarter of 2012 from 25% in 2011, with an associated decrease in the volume of antibiotics sold (Figure 2). Data released by the Chinese MOH indicates that the percentage of prescriptions for antimicrobials decreased from 68% to 58% for hospitalized patients and from 25% to 15% for outpatients. Antimicrobial prophylaxis use associated with clean surgical procedures also decreased from 95% to 58% of cases, with the duration of use shortened from 5 days to less than 48 hours. For example, antibiotic procurement in Tianjin City was 200 million RMB Yuan lower in 2011 than in 2010, although the numbers of outpatient visits, hospitalized patients, and operations increased by 1.4 million, 84,000, and 58,000, respectively [15].

To ensure sustainable progress towards the rational use of antimicrobial agents in healthcare settings, “administrative regulations for the clinical use of antimicrobials” were issued in China in August 2012, in which the concept of “antibiotic stewardship” was integrated into the measures outlined during the special campaign [16]. The legislation considers long-term antibiotic stewardship in Chinese healthcare institutions and is expected to change the current situation in which rational antimicrobial use simply relies on incidental administrative motivation. This “administrative antibiotic stewardship” regulation is implemented by the Chinese MOH. They may also provide a valuable reference for other countries with similar healthcare systems [17].

## Further Problems for the Containment of AMR

Although the special campaign launched by the MOH to promote the rational use of antibiotics in healthcare settings has had some success, several factors need to be considered to establish a long-term and nationwide framework for sustainable AMR containment in China. Firstly, several ministries share regulatory oversight of antimicrobial use. The State Food and Drug Administration is responsible for the registration, production, quality control, and distribution of antimicrobials. The Ministry of Agriculture is independently responsible for complete oversight of antibiotic use in animal feed and veterinary care. The MOH exclusively administers the use of antibiotics in healthcare settings. This power-sharing administrative system has a tendency to dilute direct responsibility. New mechanisms should be explored to provide integrated policies to promote the rational use of antibiotics and AMR containment in related fields. Secondly, medical school curricula and continuing medical education should be improved. At present, most medical students in China do not study the relevance of antibiotic therapy to AMR. Thirdly, public education should be provided to ensure that the general public are aware of the risks associated with AMR, which will likely help to reduce over-the-counter sales and self-medication of antibiotics. Finally, and critically, the government should widen the healthcare reforms and provide financial guarantees to medical institutions to ensure that economic incentives from drug sales are eliminated. This will return healthcare institutions to a not-for-profit status and aid professional standards [9],[12],[18],[19].

## Abbreviations

AMR: antimicrobial resistance
MOH: Ministry of Health
Mohcas: Chinese Ministry of Health Center for Antibacterial Surveillance
Mohnarin: Chinese Ministry of Health National Antimicrobial Resistance Investigation Net
MRSA: methicillin-resistant *Staphylococcus aureus*
